# Left bundle fascicular versus left bundle trunk pacing: A comparison of their electrical synchrony parameters

**DOI:** 10.1016/j.ipej.2024.07.006

**Published:** 2024-07-30

**Authors:** Álvaro Estévez Paniagua, Sem Briongos-Figuero, Ana Sánchez Hernández, Roberto Muñoz-Aguilera

**Affiliations:** Cardiology Department. Infanta Leonor Hospital, Gran Vía Del Este, 28030, Madrid, Spain

**Keywords:** Left bundle branch capture, Left bundle trunk pacing, Left bundle fascicular pacing, Left bundle branch pacing criteria

## Abstract

**Background:**

Variation in human left bundle branch (LBB) anatomy has a significant effect on the sequence of left ventricular depolarization. However, little is known regarding the electrophysiological characteristics of pacing different LBB fascicles.

**Objective:**

We aimed to analyse the different electrocardiographic characteristics of LBB pacing (LBBP) attending to the site of pacing at the LBB system.

**Methods:**

In 200 consecutive patients with confirmed LBBP, we distinguished left bundle trunk capture (LBTP) from any LB fascicular pacing (LBFP) based on the presence of LB potentials and paced QRS morphologies. We compared them regarding procedure, LBBP criteria and electrical synchrony parameters.

**Results:**

One hundred and seventy-three patients with LBFP were compared to 25 patients with LBTP. Left septal and posterior fascicles were significantly more prevalent than left anterior in LBFP (46.8 %, 41.0 % and 12.2 % respectively). QRS transition criteria (80.0 % vs 61.8 %; p = 0.077), selective LBBP (40.0 vs 21.5 %; p = 0.101), paced QRS width (110.3 ± 16.8 ms vs 115.4 ± 14.9 ms; p = 0.117), V6-RWPT (79.2 ± 10.7 ms vs 75.3 ± 9.7 ms; p = 0.068) and interpeak interval (42.5 ± 19.1 ms vs 45.7 ± 12.9 ms; p = 0.282) were not significantly different between LBTP and LBFP. All short-term complications occurred in LBFP, mainly driven by septal perforations (n = 23), without any difference in the pacing parameters. Among the LBFP subgroups, only aVL-RWPT was longer when the posterior fascicle was paced.

**Conclusions:**

LBFP is much more prevalent than LBTP in unselected consecutive patients with LBBP. LBFP seems more feasible, and as good as LBTP in terms of electrical synchrony and pacing safety.

## 1 Introduction

Left bundle branch (LBB) area pacing (LBBAP) has emerged as a new option to provide physiological ventricular pacing and has been widely adopted in the last five years [1,2]. Recommendations for its use have been currently established in antibradycardia and heart failure indications [3]. Initially, two different types of capture were described during LBBAP: left ventricular septal (LVS) myocardial capture or direct LBB capture via non-selective (ns)-LBB or selective (s)-LBB pacing (LBBP) [4]. More recently, pacing any of the fascicles of the LBB has been also recognised as a type of LBBAP, distinct from LBB trunk pacing [5]. However, little is known regarding the electrophysiological characteristics of pacing different fascicles of the LBB [6], although considerable variation in human LBB anatomy has a significant effect on the sequence of left ventricular depolarization and in turn on QRS morphologies [7]. The criteria to consider which branch of the LBB is being paced were theoretically described in the MELOS study: the presence of LBB or fascicular Purkinje potentials were considered, besides the axis of the stimulated QRS, to classify whether there was LBB main trunk (LBTP) or left bundle fascicle pacing (LBFP) [8]. Other small series only had into account the paced QRS complex in the frontal plane [6,9]. Moreover, there is some discrepancy between the number of LBB fascicles: in the MELOS registry, anterior, posterior and septal fascicles were considered, meanwhile other papers did not recognise the septal fascicle. Yet, previous anatomical observations described LBB system distributions with a septal fiber or a fanlike structure. A left septal fiber arising from the posterior division or less frequently from the anterior division is common in human hearts [7,10]. Thus, use of the binary classification for the LBFP classification might seem suboptimal.

The present study aimed to analyse the different electrocardiographic characteristics of LBBP attending to the site of pacing at the LBB system. We hypothesized that, despite of the QRS axis differences between the distinct sites of pacing the LBB, there should not be a significant difference in the main parameters of electrical synchrony, having into account that, in all cases, the left ventricle activation occurs through the physiological conduction system.

## 2 Methods

### 2.1 Study design

This study enrolled all consecutive patients with an attempt of LBBAP procedure for bradycardia and/or heart failure indications (as bailed-out strategy) from February 2020 to March 2023 at our institution. Baseline clinical data and procedure-related data were acquired in a prospective way.

In those patients with confirmed LBB capture, we distinguished LBTP from any LB fascicular pacing based on the presence of LBB or fascicular Purkinje potentials and the paced QRS morphologies, whenever it was possible. Those cases without a proper ECG tracing in the frontal leads were excluded from the final analysis. We compared LBTP versus LBFP groups, attending to possible differences in the main parameters of electrical synchrony: paced QRS duration, paced V6-RWPT, paced V1-RWPT and V1-V6 interpeak interval were used for the comparison [11,12], along with the paced aVL-RWPT and LBBP score recently described by us [13].

The study adhered to the Helsinki Declaration as revised in 2013, and the Institutional Bioethical Committee approved the research protocol. All patients were informed about the nature of the conduction system pacing device and provided informed consent.

### 2.2 Procedure description

In our laboratory the LBBAP implantation procedure is routinely performed using the “single lead technique” [1,14,15] by using Medtronic C315His sheath and 3830-69 lumenless active fixed helix screw-in lead, or Boston Scientific SSCP sheaths and stylet-driven Ingevity or Fineline leads.

Procedures were recorded on a digital electrophysiological system (General Electric, USA). The measurements were performed using all 12 surface ECG leads and the endocardial channel recorded simultaneously, digital callipers and fast sweep speed (100 mm/s). At least three QRS complexes were measured, and the values were averaged.

### 2.3 Left bundle branch capture criteria

Direct LBB capture was considered if unipolar paced QRS morphology in lead V1 showed right bundle branch (RBB) conduction delay pattern and at least one of the following.

(a) Transition from ns-LBBP to s-LBBP during decrease in pacing output, characterized by distinct isoelectric interval before the local electrogram (EGM) and the appearance of M/rSR’ pattern and wide R′ with a notch in lead V1, S wave in V5/V6, with constant V6-RWPT.
(b) Transition from ns-LBBP to LVS capture defined by an abrupt prolongation of V6-RWPT≥10 ms during decrease in pacing output [16].

When the above QRS transition criteria were not achieved, we used the following criteria to diagnose LBB capture.

(c) Transition from LVS to ns-LBBP during the lead screwing-in (defined as a sudden shortening of V6-RWPT by > 10 ms seen during mid to deep septal lead progression at the same target, between two consecutive pacing manoeuvres, deep and deeper, with a QR/rSR’ pattern in V1 in both, and V6-RWPT remaining short and constant at high and low output in the deeper final position) [17].
(d) Accomplishment of the novel ECG-based combined criterion of either V6-RWPT <75 ms or V6-V1 interpeak interval ≥33 ms [12].

### 2.4 Left bundle system pacing site criteria

The criteria to determine the site of pacing were based on a combination of previous studies of left bundle fascicular block and those used in the MELOS study [6,8,9].

**Fig. 1:**
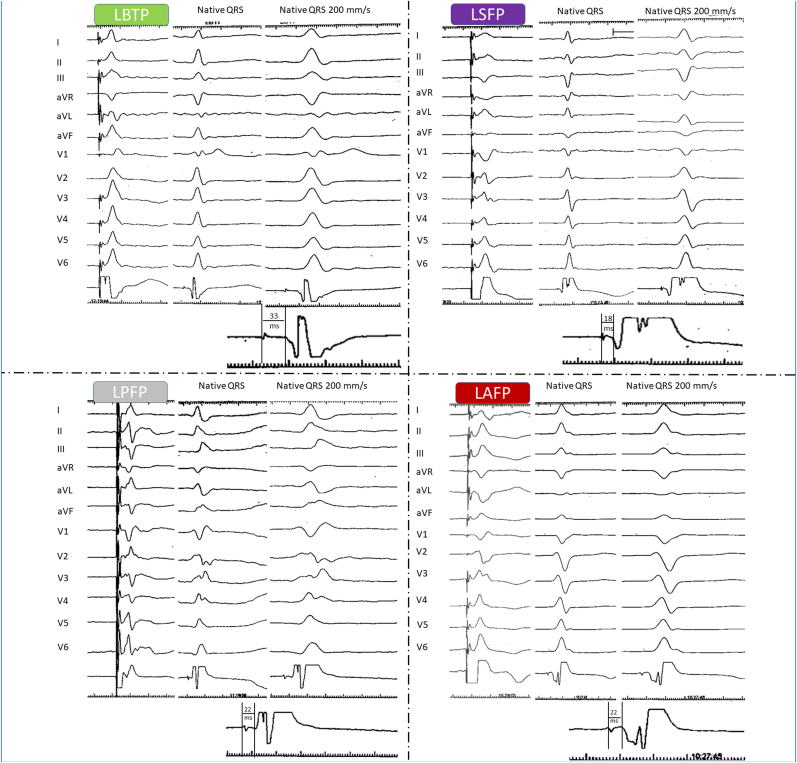
**Sites of LB system pacing: Paced QRS and EGM** LBTP: the paced QRS shows a similar left ventricular electrical activation compared to the native QRS, with a LB potential-vEGM Interval between 25 and 35 ms; LSFP: the paced QRS shows predominant R wave in lead II and negative component in lead III. Compared to the native QRS, the paced left ventricular activation is also similar, although the Purkinje potential-vEGM Interval is < 25 ms; LPFP: the paced QRS is predominantly negative in the inferior leads, with left axis in the frontal plane, different from the native QRS. The Purkinje potential-vEGM Interval is < 25 ms; LAFP: the paced QRS is predominantly negative in I and aVL leads, and positive in the inferior leads, with right axis in the frontal plane. The Purkinje potential-vEGM Interval is < 25 ms. LBTP: left bundle trunk pacing; LBFP: left bundle fascicular pacing; LSFP: left septal fascicle pacing; LPFP: left posterior fascicle pacing; LAFP: left anterior fascicle pacing.

The presence of LBB or fascicular Purkinje potentials were analysed, and the potential-ventricular electrogram (vEGM) interval was measured, all together with the QRS axis at the frontal plane. The different types of pacing sites were considered as following (Fig. 1).

• Left Bundle trunk pacing (LBTP): potential-vEGM interval of 25–35 ms and QRS axis similar to sinus rhythm.
• Left anterior fascicular pacing (LAFP): dominant S wave in leads I and aVL, dominant R wave in inferior leads, right-axis deviation, with potential-vEGM interval <25 ms or without potential.
• Left septal fascicular pacing (LSFP): potential-vEGM interval <25 ms or without potential and QRS axis like sinus rhythm or inferior axis with negative component in lead III.
• Left posterior fascicular pacing (LPFP): dominant R wave in leads I and aVL, dominant S wave in inferior leads, left-axis deviation, with potential-vEGM interval <25 ms or without potential.

### 2.5 Measurements

In each patient, every available paced QRS type (s-LBB, ns-LBB and LVSP) and native QRS were measured. The following QRS characteristics were obtained.

(1) Native and paced QRS duration measured from the earliest onset (and from the stimulus for paced QRS) in any of the 12 EC G leads recorded simultaneously.
(2) RWPT, measured from the stimulus to the peak of R wave in lead V1, V6 and aVL.
(3) V6-V1 interpeak interval, measured from the R-wave peak in lead V6 to the R-wave peak in lead V1 during simultaneous recording.

### 2.6 Statistical analysis

Continuous variables were expressed as mean ± standard deviation (SD) and categorical data as numbers or percentages. Continuous variables were compared using the Student *t*-test or the Mann-Whitney *U* test, as appropriate. Categorical variables were compared using χ^2^, or the Fisher exact test when the conditions required for the former test were not met. For paired comparisons, Student *t*-test was used for Gaussian variables, Wilcoxon non-parametric test for non-Gaussian variables and McNemar-Broker test for categorical variables. The data managements and analyses were performed with SPSS, version 20.0 (IBM corporation, Chicago, Illinois). Significance was defined as p < 0.05.

## 3 Results

**Fig. 2:**
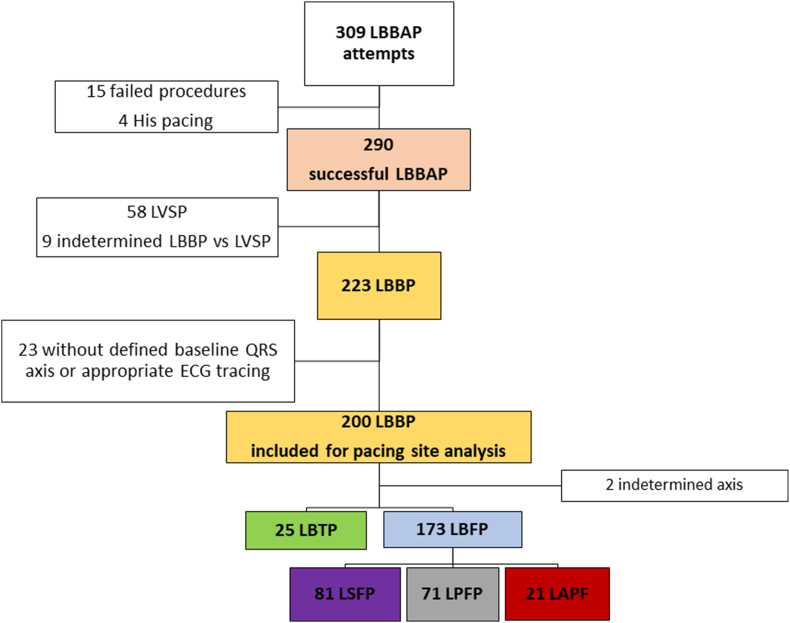
**Study population enrolment flowchart.** LBBAP: left bundle branch área pacing; LVSP: left ventricle septal pacing; LBBP: left bundle branch pacing; LBTP: left bundle trunk pacing; LBFP: left bundle fascicular pacing; LSFP: left septal fascicle pacing; LPFP: left posterior fascicle pacing; LAFP: left anterior fascicle pacing.

**Fig. 3:**
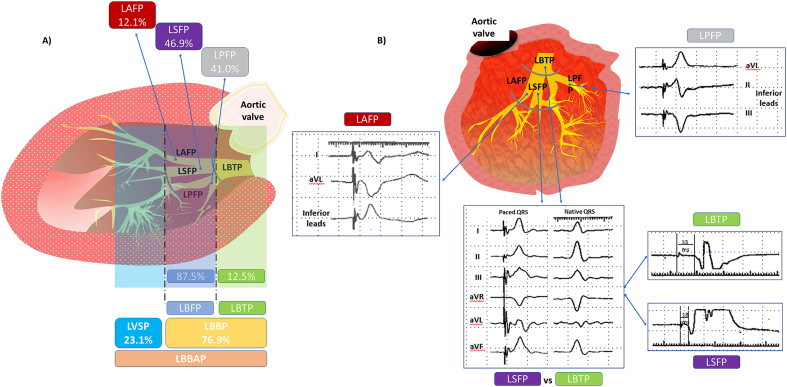
**Left bundle branch area sites of pacing and LBFP classification.** A) Sagital left ventricle dissection. Percentages show the prevalence of the different types of LBBAP in the study population B) Left ventricle open-book dissection. Frontal axis QRS morphology and left bundle/Purkinje potentials criteria used to distinguish LBTP from LBFP, and between different types of fascicular pacing are depicted. LAFP is characterized by right and inferior axis in the frontal plane, with rS pattern in I and aVL leads. LPFP shows left and superior frontal axis, with rS pattern in inferior leads (usually R in lead II > R in lead III). LBTP and LSPF usually have paced QRS axis similar to that of native QRS; the difference between them depends on the presence or absence of LB/Purkinje potentials. LB potential (25–35 ms to v-EGM) is considered mandatory to define LBTP. Conversely, fascicular/Purkinje potential (0–25 ms to v-EGM) are not necessary to consider LBFP. Abbreviations as in Figs. 1 and 2.

A total of 309 patients with intended LBBAP were screened (Fig. 2). Successful LBBAP was accomplished in 93.8 % of procedures, with direct LBB capture in 223 patients (76.9 %). QRS transition criteria were demonstrated in 140 out of 290 successful procedures (48.3 %) and in 17 patients both QRS transitions (from ns-LBBP to s-LBBP and from LVSP to ns-LBBP) were observed. The final study population consisted of 200 patients who accomplished LBB capture and had an appropriate frontal leads ECG tracing. Two cases did not have any of the previously mentioned criteria to determine the site of pacing and were considered indetermined morphology. LBFP was the most frequent type of pacing (86.5 %), with a majority of left septal and posterior fascicular pacing (40.5 % and 35.5 %, respectively), meanwhile LBTP was present only in 12.5 % of cases (Fig. 3).

### 3.1 Baseline characteristics

Table 1 and Supplemental Table 1 show the baseline characteristics of the study population according to the site of pacing (LBTP vs LBFP, and among LBFP subgroups, respectively). Majority of patients received a device due to bradycardia pacing indications (95.5 %) and only 8.7 % of patients presented with left ventricular ejection fraction below 40 %. Non-diseased LBB (narrow QRS complex or isolated right bundle branch block (RBBB)) was present in 65.5 % of patients and 12.0 % (n = 24) of patients showed complete LBB block (LBBB). There were no significant differences in baseline characteristics between LBTP and LBFP, especially regarding left ventricle size and function, interventricular septum (IVS) thickness and atrial volume. Only baseline QRS width was significantly longer in the LBFP group.

**Table 1:** Baseline characteristics according to the type of pacing location: LBTP vs. LBFP.

	LBTP (n = 25)	LBFP (n = 173)	p value
**Clinical variables**			
• Age (years)	80.1 ± 11.6	77.9 ± 10.2	0.340
•BMI	30.3 ± 5.1	28.4 ± 5.0	0.087
•Male	12 (48.0)	90 (52.0)	0.831
•Hypertension	22 (88.0)	142 (82,6)	0.774
•Diabetes mellitus	6 (24.0)	51 (29.7)	0.643
•AF	11 (44.0)	77 (44.8)	1.000
•CKD^a^	4 (16.0)	31 (18.1)	1.000
•Coronary heart disease	4 (16.0)	14 (8.1)	0.255
•COPD	4 (16.0)	15 (8.7)	0.273
•Previous heart failure	4 (16.0)	42 (24.4)	0.453
**Pacing indication**			0.777
•AV block rowhead	12 (48.0)	65 (37.6)	
•Slow AF/bradycardia-tachycardia syndrome	6 (24.0)	70 (40.5)	
•Sinus node disease	5 (20.0)	19 (10.9)	
•CRT	1 (4.0)	8 (4.6)	
•Bifascicular block + syncope/alternant BBB	1 (4.0)	8 (4.6)	
**Echocardiographic parameters**			
•LVEF (%)	58.8 ± 11.5	57.7 ± 9.7	0.617
•LVEF <40 %	3 (12.0)	14 (8.3)	0.467
•LVEDD (mm)	45.5 ± 7.5	45.4 ± 6.4	0.936
•IVS thickness (mm)	11.7 ± 2.4	12.1 ± 2.4	0.376
•Left atrial volume (ml/m2)	44.4 ± 15.0	45.5 ± 20.9	0.816
**Baseline ECG characteristics**			
•PR interval	177.3 ± 76.1	196.9 ± 62.6	0.394
•Native QRS width (ms)	103.2 ± 20.5	119.2 ± 31.4	**0.014**
•QTc interval	440.6 ± 34.0	437.9 ± 33.9	0.778
•Wide QRS complex (>120 ms)	6 (24.0)	79 (45.7)	0.051
**Baseline ECG morphology**^b^			0.299
•Isolated RBBB	2 (25.0)	26 (29.5)	
•RBBB + LFB	2 (25.0)	26 (29.5)	
•LBBB	1 (12.5)	17 (19.3)	
•Isolated LAFB	2 (25.0)	7 (8.0)	
•NIVCD	1 (12.5)	2 (2.3)	
•Asystole/PM dependent	0 (0.0)	10 (11.4)	

Values are mean ± standard deviation (SD) and n (%).

LBTP: left bundle trunk pacing; LBFP: left bundle fascicular pacing; AF: atrial fibrillation; AV: atrioventricular; BBB: bundle branch block; BMI: body mass index; CKD: chronic kidney disease; COPD: chronic obstructive pulmonary disease; CRT: cardiac resynchronization therapy; LVEF: left ventricular ejection fraction; LVEDD: Left ventricular end-diastolic diameter; IVS: interventricular septum; RBBB: right bundle branch block; LFB: left fascicular block; LBBB: left bundle branch block; LAFB: left anterior fascicular block; NIVCD: non-specific intraventricular conduction disease: PM: pacemaker.

a Glomerular filtration rate <60 ml/kg/1.73 m^2^

b Percentages related to patients with conduction system disease.

### 3.2 Procedural and electrical parameters

Table 2 and Supplemental Table 2 display data related to the implantation procedures depending on the site of pacing. Most procedures were performed using Medtronic 3830-69 leads. LBTP trended to be more frequently achieved with lumenless leads. Conversely, stylet-driven leads were more prevalent in the LPFP and LAFP subgroups. The time of LBBP lead implantation (from sheath introduction to sheath removal) was similar between LBTP and LBFP, and not significantly longer in the LPFP subgroup. There was a trend towards shorter paced QRS duration in the LBTP group compared to the LBFP group (110.3 ± 16.8 ms vs 115.4 ± 14.9 ms, respectively; p = 0.117). Left conduction system potential was found in 93 cases (45.5 %). Purkinje potentials were more frequent, although not significantly, in the septal fascicle pacing group than in the other two fascicles (44.3 % vs 36.3 %; p = 0.181), with no difference in the potential-vEGM interval among them, and longer interval in the LBTP group, as defined before in the MELOS criterion (28.1 ± 3.0 ms in the LBTP group vs 18.0 ± 3.9 ms in the LBFP group; p < 0.001). Related to the functional electrical parameters, there were no differences between both groups, neither in the threshold and impedance, nor in the R wave detection. Also, when comparing LBFP subgroups, no differences were found in pacing parameters.

**Table 2:** Comparison of procedural characteristics according to the type of pacing location: LBTP vs. LBFP.

	LBTP (n = 25)	LBFP (n = 173)	p value
**LBBAP lead**			0.136
**•Medtronic 3830**–**69 lumenless lead**	25 (100.0)	155 (89.6)	
**•Boston Scientific stylet-driven leads**	0 (0.0)	18 (10.4)	
**LBBAP lead placement**			
•Fluoroscopy (min)	8.3 ± 9.1	8.9 ± 9.7	0.813
•Procedure (min)	18.4 ± 17.9	19.9 ± 18.4	0.733
**Paced QRS width (ns-LBBP)**			
•QRS duration from onset (ms)	110.3 ± 16.8	115.4 ± 14.9	0.117
•QRS duration from stimulus (ms)	147.8 ± 17.3	147.8 ± 18.2	0.990
LB potential	25 (100.0)	68 (40.0)	**<0.001**
LB potential to v-EGM onset (ms)	28.1 ± 3.0	18.0 ± 3.9	**<0.001**
Acute pacing parameters			
•R wave sensing (mV)	8.7 ± 4.6	9.8 ± 4.6	0.295
•Impedance (Ohm; unipolar)	949.4 ± 209.4	959.9 ± 220.2	0.824
•Threshold (Volts x 0.4 ms; unipolar)	0.88 ± 0.39	0.85 ± 0.48	0.776
**Type of device implanted**			0.578
•**SR**	8 (32.0)	42 (24.3)	
•**DR**	17 (68.0)	121 (69.9)	
•**CRT-P**	0 (0.0)	3 (1.8)	
•**CRT-ICD**	0 (0.0)	7 (4.0)	

Values are mean standard deviation (SD) and n (%).

LBTP: left bundle trunk pacing; LBFP: left bundle fascicular pacing; LBBAP: left bundle branch area pacing; ns-LBBP: non-selective left bundle branch pacing; SR: single chamber pacemaker; DR: dual chamber pacemaker; CRT-P: cardiac resynchronization therapy-pacemaker; CRT-ICD: cardiac resynchronization therapy-implantable cardioverter defibrillator; LB: left bundle; v-EGM: ventricular electrogram.

### 3.3 Comparison of LBBP criteria depending on the site of pacing

Table 3 and Supplemental Table 3 depict data related to the LBBP criteria depending on the site of pacing. Transition criteria trended to be more frequent in LBTP than in LBFP (80.0 % vs 61.8 %, respectively; p = 0.077). All the QRS morphology changes occurred during decremental pacing manoeuvres in LBTP, meanwhile in LBFP the transition was seen during the lead screwing-in and interrupted pacemapping in 26.2 % of the patients (p = 0.007). When transition from non-selective LBBP to LVSP occurred, the abrupt lengthening in V6-RWPT was longer in the LBTP group (20.6 ± 10.1 ms vs 16.1 ± 4.3 ms; p = 0.006). Also, there was a trend towards more selective capture achievement in LBTP (40.0 % vs 22.5 %; p = 0.101). In LBFP subgroups, LSFP showed higher rates of transition criteria consecution, transition during pacing manoeuvres and selective capture achievement.

**Table 3:** LBBP characteristics according to the type of pacing location: LBTP vs. LBFP.

	LBTP (n = 25)	LBFP (n = 173)	p value
R' wave in lead V1	25 (100.0)	171 (98.8)	1.000
**Ventricle activation parameters**			
V6-RWPT (ms)	79.2 ± 10.7	75.3 ± 9.7	0.068
aVL-RWPT (ms)	76.5 ± 15.1	80.5 ± 13.4	0.244
V1-RWPT (ms)	119.2 ± 15.6	121.0 ± 14.0	0.557
Interpeak interval (ms)	42.5 ± 19.1	45.7 ± 12.9	0.282
**Transition criteria**	20 (80.0)	107 (61.8)	0.077
**Type of transition**			0.085
•NS-LBBP to LVSP	10 (50.0)	68 (63.5))	
•NS-LBBP to S-LBBP	5 (25.0)	27 (25.3)	
•Both	5 (25.0)	12 (11.2)	
**Moment of transition**			**0.007**
•During pacing manoeuvres	20 (100.0)	79 (73.8)	
•During lead screwing-in	0 (0.0)	28 (26.2)	
S-LBBP capture	10 (40.0)	39 (22.5)	0.101
ΔV6-RWPT NS-LBBP to LVSP (ms)	20.6 ± 10.1	16.1 ± 4.3	**0.006**
**Combined criteria**			
V6-RWPT<75 ms or V6V1-RWPT>33 ms	21 (84.0)	158 (91.3)	0.271
LBBP score	4.7 ± 2.1	5.0 ± 1.9	0.637

Values are mean ± standard deviation (SD) and n (%).

RWPT: R wave peak time; NS-LBBP: non-selective left bundle branch pacing; LVSP: left ventricle septal pacing; S-LBBP: selective left bundle branch pacing; ΔV6-RWPT NS-LBBP to LVSP: V6-RWPT shortening between the transition of NS-LBBP to LVSP.

Attending to the ECG-based criteria, no significant differences were found between LBTP and LBFP in paced V6-RWPT, aVL-RWPT and V6-V1-RWPT interval, although a trend towards shorter V6-RWPT in LBFP was found (75.3 ± 9.7 ms vs 79.2 ± 10.7 ms; p = 0.068). Neither there were any significant differences between LBFP subgroups regarding their ECG-based criteria values, apart from aVL-RWPT, that was shorter in LAFP and longer in LPFP.

Finally, we analysed the accomplishment of combined criteria: there were no differences in the consecution of V6RWPT<75 ms or interpeak interval>33 ms. Meanwhile, the LBBP score did not show any difference between LBTP and LBFP (4.7 ± 2.1 vs 5.0 ± 1.9 respectively; p = 0.637), but anterior fascicular pacing showed higher LBBP score than posterior and septal pacing (6.9 ± 1.4 vs 4.7 ± 1.9 vs 5.0 ± 1.9, respectively; p = 0.004).

### 3.4 Comparison of morphological characteristics

We observed that R wave transition in precordial leads occurred in V3/V4 leads in majority of LBTP (80 %) and it appeared significantly more often in V5/V6 leads among LBFP compared to LBTP (45.4 % vs 20 %, respectively; p = 0.016). Among LBFP, R wave transition also appeared significantly later (V5/V6) among LPFP compared to LSFP or LAFP (56.3 % vs 37 % vs 38.1 %, respectively; p = 0.046). Besides, in 125 cases (62.5 %) the final LBBP morphology exhibited an S wave in V6 lead. This finding was more frequently seen in LBFP compared to LBTP (66.1 % vs 44 %, respectively; p = 0.032), and tended to occur more often when pacing the left posterior fascicle (74.6 % vs 59.3 % vs 61.9 % for LPFP, LSFP and LFAP, respectively; p = 0.125). No differences were found between LBTP and LBFP regarding the amplitude of the S wave (0.24 ± 0.14 mV for LBTP vs 0.25 ± 0.21 mV for LBFP, p = 0.760) neither among patients with LBFP, although a tendency was found to a deeper S wave in LPFP and LAFP compared to LSFP (0.28 ± 0.22 mV and 0.29 ± 0.27 mV vs 0.21 ± 0.17 mV, respectively; p = 0.107 (for LSFP vs LPFP) and p = 0.271 (for LSFP vs LAFP)).

## 4 Short-term complications

There was an episode of ventricular fibrillation (VF) during lead penetration, solved with defibrillation, without further consequences. Complications rate at 30-days was 16.5 % (n = 33), mainly driven by septal perforation during the implant (n = 23), followed by acute pacing threshold >2 V × 0.4 ms (n = 5), pneumothorax (n = 2), lead dislodgement (n = 1), acute chest pain (n = 1) and the previously mentioned VF. The rate of intraprocedural lead dislodgement was significantly higher with stylet driven leads compared to lumenless (p = 0.030) as it was the percentage of septal perforations (p < 0.001). No stroke or other thromboembolic complications were observed in cases of perforations of the lead into the LV cavity. Lead repositioning was feasible in all cases of septal perforation or lead dislodgement without further complications. No deaths, infection or cardiac perforation were observed at 30-days after the implant. When comparing both study groups, all the acute issues happened in the LBFP group, without anyone found in the LBTP group (17.0 % vs 0.0 % complications rate, respectively; p = 0.030). Most of these issues occurred in the posterior fascicle pacing group (n = 21, 29.6 % complications rate, vs 4.8 % in the anterior fascicle group and 8.9 % in the septal fascicle group; p = 0.001).

## 5 Discussion

### 5.1 Comparison of LBTP versus LBFP

Our results are quite consistent with those of MELOS study [8], in terms of higher feasibility in achieving successful LBBP with LB fascicular pacing rather than LB trunk pacing. Also, they support the similarity between LBTP and LBFP in terms of electrical synchrony, as there were not significant differences either in the paced QRS duration or in ventricular activation parameters (V6-RWPT, aVL-RWPT and V6-V1 RWPT Interpeak Interval). Although there was a trend for shorter paced QRS duration in the LBTP group, it could be explained by the tendency to longer native QRS duration in the LBFP group, which might reflect a more diseased conduction system, and thus, an additional barrier to narrow the QRS.

There is some controversy about the electrical synchrony behaviour attending to the LBB system pacing location: first analysis by Lin J et al. showed no significant differences between LBTP and LBFP groups in the main ECG parameters of paced ventricular activation, nor in the global paced QRS duration [9]. Contrarily, Liu X et al. found that LBTP had shorter paced QRS and smaller paced QRS areas than LBFP, although left ventricular activation time did not differ [6]. To add more difficulty, recently the MELOS study described that QRS duration was shorter in the LBFP group and seemed to offer faster activation of the LV than LBTP, as suggested by shorter paced V6-RWPT [8]. Theoretically, LBTP should result in better cardiac synchrony by sequentially activating the left ventricle (LV) [18]. In contrast, LBFP would lead to different ventricular activation sequences, thus resulting in impaired left ventricular synchrony [6]. However, it has been hypothesized that, when pacing the fascicles, breakout from the conduction system would occur more rapidly and this would limit the amount of adjacent myocardium that is activated. It has been described that pacing the LB system more distally provokes faster LV activation, although more delayed right ventricle activation [19]. Moreover, the subendocardial interconnections between fascicles on the upper two-thirds of the IVS might outbreak fast anterograde conduction to the Purkinje network and subsequently to the ventricular myocardium, hypothetically correcting the potential delay of a different axis activation sequence. Thus, our finding of a trend towards shorter V6-RWPT in the LBFP group would be concordant with these mechanisms of faster LV depolarization.

LBB selective capture trended to be more prevalent with LB trunk pacing, probably indicating that the presence of Purkinje potential, considered mandatory by Huang et al. for the LBTP definition [1], represents a lead closer to the conduction system, and thus, easier to obtain transition from non-selective to selective LBB stimulation. Also, the more isolated fibers of LB trunk could play a role. Similarly, transition phenomena were much more frequent in the LBTP group, seen during decremental pacing manoeuvres, also suggesting a deeper lead location into the septum, with transition during lead screwing-in more frequently found in the LBFP group, with less common finding of Purkinje potentials in this group.

### 5.2 Comparison of LBFP subgroups

The existence of septal fascicular pacing was supported in our paper by two points: 1) paced QRS morphologies that did not accomplish either the definition of LBTP (absence of Purkinje potential or Purkinje potential-vEGM interval less than 25 ms) or the common anterior or posterior fascicular pacing axis, and 2) showed a similar axis to the native QRS.

All the ventricular activation parameters (V6-RWPT, V1-RWPT, V6V1-RWPT interpeak interval) did not differ among the three fascicles, except for aVL-RWPT, that was longer in the posterior fascicle pacing subgroup. Also, no significant differences were found in the QRS duration among the subgroups. This could be related to the Purkinje network that connects the different divisions of the LBB and might allow a rapid and more homogeneous left ventricle depolarization, despite the different initial electrical forces, all along with a similar right ventricle activation through the septum in non-selective capture. The referred differences in the initial depolarization could be the reason for longer RWPT in aVL lead in the posterior fascicle pacing group, reflecting the common left axis deviation at the frontal plane of the resulting left anterior fascicular block morphology, as well as the shorter aVL-RWPT with the anterior fascicle pacing, due to the paced left posterior fascicular block morphology, with characteristic Rs and rS patterns respectively in the aVL lead.

Nevertheless, although no differences in most ECG-based criteria were found among the fascicular pacing subgroups, transition criteria, especially during decremental pacing manoeuvres, as well as LBB capture, were more frequent when septal fascicle was paced. It does not seem to be related to the lead position, as the Purkinje potential-vEGM interval was similar between all fascicles subgroups. Otherwise, this finding is like that of LBTP compared to the whole group of LBFP, and it might suggest that septal fascicle behaves as an extension of the trunk of the left bundle branch. Also, although not significant, Purkinje potentials were slightly more frequent in the septal fascicle pacing maybe indicating a little deeper lead location into the septum, closer to the conduction system and, thus, being easier to show transition phenomena during threshold test and selective capture, in the same way as LBTP.

### 5.3 Morphological characteristics

LBFP and LBTP showed differential morphological characteristics as R wave transition in precordial leads occurred later, in V5/V6 leads, and S wave appeared more often in LBFP compared to LBTP. However, in most of LBTP cases, R wave transition occurred in V3/V4 leads and S wave was less frequently seen. As expected, distal pacing within the LBB results in earlier activation of the left lateral wall and more delayed activation of the right ventricle, which is translated into a poorer growth of the R wave in precordial leads and into a more prevalent and prominent S wave. Furthermore, proximal pacing should result in earlier activation of the right ventricle as retrograde conduction through the His-Purkinje network is faster. Although our findings are congruent with the current definition of left bundle fascicle pacing [8], we should take into account that the final characteristics of the paced QRS complex are highly influenced by capture selectivity and baseline QRS morphology [19].

### 5.4 Procedure comparison: short-term complications and pacing parameters

We did not find a difference neither in the procedural duration nor in the fluoroscopy time between LBTP and LBFP groups. Among the LBFP subgroups, posterior fascicle pacing showed longer fluoroscopy and procedural durations. Paradoxically, it has been described an easier access to posterior fascicle positions, due to less isolation of these fibers when compared with the LBB trunk, and sheath more easily placed in the mid or mid-inferior septum [8,20]. We hypothesize that this finding might be caused by two factors: first, we found an objective higher rate of septum perforations in this location, that could have delayed the final success of the lead deployment; second, hypothetically, it could be related with the choice of this position as a bail-out strategy after a failure in achieving LBBP when targeting the LBB trunk. This could be sustained by the fact that LBTP was much more infrequent than LBFP as final position, probably indicating more plausible success in the former group.

Overall short-term complications only occurred in the LBFP group, most of them driven by LBBAP technique issues, mainly septal perforations in the posterior fascicle group. This might be related to several factors: although there was not any difference in IVS thickness between both LBTP and LBFP groups, this measurement corresponds to the maximum width, usually found in the basal anterior septum. It is also known that IVS is often narrower in mid or mid-inferior regions, in opposition to thicker basal septum, especially in elderly populations with hypertension [21] like ours, so it could be easier to protrude into the LV cavity during the lead screwing-in when targeting posterior fascicle. Moreover, it has already been discussed that QRS transition phenomena [16], were less frequent in the LBFP group. This lack of gold-standard demonstration of LBBP could have provoked a deeper penetration into the septum, with the consequent higher risk of perforation. Nevertheless, lead repositioning was feasible in all cases of septal perforation or lead dislodgement. Thus, despite of the higher rate of acute complications related to the LBBAP technique in the LBFP group, safety in terms of acute pacing parameters was similar between both groups.

### 5.5 Limitations

This study was conducted in a single center. The QRS morphology analysis to determine the site of pacing was done with the ECG tracings recorded during the lead implantation, without the ability to measure the exact QRS axis. Nevertheless, the previous definitions of LBFP have been also based in QRS patterns analysis, with or without Purkinje potentials determination, rather than vectorial measurements [6,8,9]. Another pitfall of the study would be the lack of anatomical information about the lead tip position. However, we consider this hard to stablish during the implant: it would probably require a second lead in the His position, or right ventriculography to mark the tricuspid valve annulus aspect. Even with this, it would only be a partial analysis of a 3D-model. Yet, previous small analysis on this issue, using fluoroscopic localization in right anterior oblique 30° projection, have already shown the expected more distal and off-centered tip location in the LBFP groups [6,9].

## 6 Conclusions

Our study supports that left bundle fascicular pacing is much more frequent than left bundle trunk pacing. It does not only seem more feasible, but as good as LBTP in terms of electrical synchrony and pacing safety, just with a little concern about higher septal perforation rate. The easier access of the sheath to slightly more distal septal positions, along with the wider and less isolated distribution of the LB fascicles, probably makes left bundle fascicular pacing a more practical way of conduction system stimulation. The recognition of the different paced QRS morphologies in the frontal axis, along with the analysis of the Purkinje potentials, arise as important tools to fully understand the whole map of the left bundle branch area pacing.

## Disclosures

A.E.P has received speaker honoraria and consulting fees from Medtronic and Boston Scientific.

S.B.F has received speaker honoraria and consulting fees from Medtronic and Boston Scientific.

All remaining authors have declared no conflicts of interest.

There were not any funding disclosures related to this original article.

## Declaration of competing interest

The authors declare the following financial interests/personal relationships which may be considered as potential competing interests.

Sem Briongos Figuero reports a relationship with Medtronic Inc that includes: consulting or advisory. Alvaro Estevez Paniagua reports a relationship with Medtronic Inc that includes: consulting or advisory. Sem Briongos Figuero reports a relationship with Boston Scientific Corp that includes: consulting or advisory. Alvaro Estevez Paniagua reports a relationship with Boston Scientific Corp that includes: consulting or advisory. If there are other authors, they declare that they have no known competing financial interests or personal relationships that could have appeared to influence the work reported in this paper.
