# Supplementary material for: Left bundle fascicular versus left bundle trunk pacing: A comparison of their electrical synchrony parameters
Source: Indian Pacing Electrophysiol J. 2024 Jul 30;24(5):239–46. doi: 10.1016/j.ipej.2024.07.006 (PMC11480846; doi:10.1016/j.ipej.2024.07.006)
Supplement: Multimedia component 1 [file mmc1.docx]

**Supplemental Table 1. Baseline characteristics according to the type of fascicular pacing location.**

|  | LSFP  (n=81) | LPFP  (n=71) | LAFP  (n=21) | p value |
| --- | --- | --- | --- | --- |
| **Clinical variables** |  |  |  |  |
| - Age (years) | 77.8±10.1 | 77.8±10.1 | 79.7±8.6 | 0.695 |
| - BMI | 28.8±5.2 | 28.3±4.6 | 27.4±5.5 | 0.540 |
| - Male | 44 (48.9) | 35 (49.3) | 11 (52.4) | 0.831 |
| - Hypertension | 71 (87.7) | 55 (78.6) | 16 (76.2) | 0.244 |
| - Diabetes mellitus | 24 (29.6) | 20 (28.6) | 7 (33.3) | 0.916 |
| - AF | 38 (46.9) | 32 (45.7) | 7 (33.3) | 0.526 |
| - CKD* | 16 (19.8) | 9 (13.0) | 6 (28.6) | 0.236 |
| - Coronary heart disease | 7 (8.6) | 6 (8.6) | 1 (4.8) | 0.833 |
| - COPD | 9 (11.1) | 6 (8.6) | 0 (0.0) | 0.274 |
| - Previous heart failure | 21 (25.9) | 16 (22.9) | 5 (23.8) | 0.906 |
| **Pacing indication** |  |  |  | 0.599 |
| - AV block | 32 (39.5) | 24 (33.8) | 9 (42.9) |  |
| - Slow AF/bradycardia-tachycardia syndrome | 32 (39.5) | 32 (45.1) | 6 (28.6) |  |
| - Sinus node disease | 8 (9.9) | 8 (11.3) | 3 (14.3) |  |
| - CRT | 3 (3.7) | 4 (5.6) | 1 (4.8) |  |
| - Bifascicular block+syncope/alternant BBB | 6 (7.4) | 3 (4.2) | 2 (9.6) |  |
| **Echocardiographic parameters** |  |  |  |  |
| - LVEF (%) | 58.1±9.1 | 57.2±10.3 | 58.3±10.0 | 0.840 |
| - LVEF < 40% | 5 (6.3) | 8 (11.8) | 1 (4.8) | 0.404 |
| - LVEDD (mm) | 44.7± 6.7 | 46.1±6.2 | 46.0± 6.0 | 0.526 |
| - IVS thickness (mm) | 11.9±2.3 | 12.2±2.5 | 13.1±2.6 | 0.152 |
| - Left atrial volume (ml/m2) | 46.6±22.5 | 45.0±20.9 | 42.7±12.6 | 0.771 |
| **Baseline ECG characteristics** |  |  |  |  |
| - PR interval (ms) | 194.3±54.5 | 191.9±63.0 | 215.4±80.6 | 0.563 |
| - Native QRS width (ms) | 119.2±30.0 | 117.2±32.8 | 126.1±32.5 | 0.520 |
| - QTc interval (ms) | 441.5±33.0 | 433.0±35.1 | 440.5±33.3 | 0.483 |
| - Wide QRS complex (>120 ms) | 39 (48.1) | 29 (40.8) | 11 (52.4) | 0.536 |
| **Baseline ECG morphology **** |  |  |  | 0.906 |
| - Isolated RBBB | 15 (35.7) | 9 (25.7) | 2 (18.2) |  |
| - RBBB + LFB | 11 (26.2) | 11 (31.5) | 4 (36.4) |  |
| - LBBB | 9 (21.4) | 6 (17.1) | 2 (18.2) |  |
| - Isolated LAFB | 2 (4.8) | 4 (11.4) | 1 (9.1) |  |
| - NIVCD | 1 (2.4) | 1 (2.9) | 0 (0.0) |  |
| - Asystole/PM dependent | 4 (9.5) | 4 (11.4) | 2 (18.2) |  |

Values are mean ± standard deviation (SD) and n (%).

*Glomerular filtration rate < 60 ml/kg/1.73 m^2^

** Percentages related to patients with conduction system disease

LBTP: left bundle trunk pacing; LBFP: left bundle fascicular pacing; AF: atrial fibrillation; AV: atrioventricular; BBB: bundle branch block; BMI: body mass index; CKD: chronic kidney disease; COPD: chronic obstructive pulmonary disease; CRT: cardiac resynchronization therapy; LVEF: left ventricular ejection fraction; LVEDD: Left ventricular end-diastolic diameter; IVS: interventricular septum; RBBB: right bundle branch block; LFB: left fascicular block; LBBB: left bundle branch block; LAFB: left anterior fascicular block; NIVCD: non-specific intraventricular conduction disease: PM: pacemaker

**Supplemental Table 2. Comparison of procedural characteristics according to the type of fascicular pacing location**

|  | LSFP  (n=81) | LPFP  (n=71) | LAFP  (n=21) | p value |
| --- | --- | --- | --- | --- |
| LBBAP lead |  |  |  | **0.006** |
| - Medtronic 3830-69 lumenless lead | 79 (97.5) | 59 (83.1) | 17 (81.0) |  |
| - Boston Scientific stylet-driven leads | 2 (2.5) | 12 (16.9) | 4 (19.0) |  |
| LBBAP lead placement |  |  |  |  |
| - Fluoroscopy (min) | 8.5±8.7 | 10.1±11.7 | 6.4±4.1 | 0.288 |
| - Procedure (min) | 18.4±15.8 | 22.8±22.6 | 15.8±8.8 | 0.229 |
| Paced QRS width (ns-LBBP) |  |  |  |  |
| - QRS duration from onset (ms) | 114.8±13.4 | 115.9±17.5 | 116.0±10.9 | 0.897 |
| - QRS duration from stimulus (ms) | 145.3±16.0 | 150.1±21.0 | 149.1±15.6 | 0.268 |
| LB potential  LB potential (bivariate analysis) | 35 (44.3)  35 (44.3) | 29 (41.4) 4 (19.0)  33 (36.3) | | 0.105  0.181* |
| LB potential to v-EGM onset (ms) | 17.9±3.7 | 17.9±4.0 | 20.0±5.0 | 0.656 |
| Acute pacing parameters |  |  |  |  |
| - R wave sensing (mV) | 9.4±3.9 | 10.0±5.3 | 10.6±4.7 | 0.510 |
| - Impedance (Ohm; unipolar) | 968.9±196.0 | 952.0±249.6 | 952.7±209.0 | 0.886 |
| - Threshold (Volts x 0.4 ms; unipolar) | 0.84±0.46 | 0.84±0.41 | 0.97±0.74 | 0.543 |
| Type of device implanted |  |  |  | 0.869 |
| - SR | 21 (25.9) | 17 (23.9) | 4 (19.0) |  |
| - DR | 54 (66.7) | 50 (70.4) | 17 (81.0) |  |
| - CRT-P | 2 (2.5) | 1 (1.4) | 0 (0.0) |  |
| - CRT-ICD | 4 (4.9) | 3 (4.3) | 0 (0.0) |  |

Values are mean ± standard deviation (SD) and n (%).

* LSFP vs LPFP/LAFP

LSFP: left septal fascicle pacing; LPFP: left posterior fascicle pacing; LAFP: left anterior fascicle pacing; LBBAP: left bundle branch area pacing; ns-LBBP: non-selective left bundle branch pacing; SR: single chamber pacemaker; DR: dual chamber pacemaker; CRT-P: cardiac resynchronization therapy-pacemaker; CRT-ICD: cardiac resynchronization therapy-implantable cardioverter defibrillator; LB: left bundle; v-EGM: ventricular electrogram

**Supplemental Table 3. LBBP characteristics according to the type of fascicular pacing location**

|  | LSFP  (n=81) | LPFP  (n=71) | LAFP  (n=21) | p value |
| --- | --- | --- | --- | --- |
| R´ wave in lead V1 | 81 (100.0) | 70 (98.6) | 20 (95.2) | 0.185 |
| Ventricle activation parameters |  |  |  |  |
| V6-RWPT (ms) | 76.1±9.4 | 74.3±10.4 | 75.1±8.6 | 0.536 |
| aVL-RWPT (ms) | 78.7±11.3 | 84.7±13.3 | 64.9±15.1 | **<0.001** |
| V1-RWPT (ms) | 121.2±13.8 | 120.3±15.0 | 122.6±11.4 | 0.781 |
| Interpeak interval (ms) | 45.0±11.8 | 46.0±15.0 | 47.4±8.5 | 0.737 |
| Transition criteria  Transition criteria (bivariate analysis) | 58 (71.6)  58 (71.6) | 39 (54.9) 10 (47.6)  49 (53.2) | | 0.163  **0.039*** |
| Type of transition   - NS-LBBP to LVSP - NS-LBBP to S-LBBP - Both | 34 (58.6)  18 (31.0)  6 (10.4) | 26 (66.7)  8 (20.5)  5 (12.8) | 8 (80.0)  1 (10.0)  1 (10.0) | 0.163 |
| Moment of transition |  |  |  | **0.013** |
| - During pacing manouvers | 48 (82.8) | 27 (69.2) | 4 (40.0) |  |
| - During lead screwing-in | 10 (17.2) | 12 (30.8) | 6 (60.0) |  |
| S-LBBP capture  S-LBBP capture (bivariate analysis) | 24 (29.6)  24 (29.6) | 13 (18.3) 2 (9.5)  15 (16.3) | | 0.111  **0.042*** |
| ΔV6-RWPT NS-LBBP to LVSP (ms) | 16.4±4.1 | 15.6±4.3 | 16.3±5.4 | 0.765 |
| Combined criteria  V6-RWPT<75 ms or V6V1-RWPT>33 ms  LBBP score | 73 (90.1)  5.0±1.9 | 64 (90.1)  4.7±1.9 | 21 (100.0)  6.9±1.4 | 0.322  **0.004** |

Values are mean ± standard deviation (SD) and n (%).

* LSFP vs LPFP/LAFP

LSFP: Left septal fascicle pacing; LPFP: Left posterior fascicle pacing; LAFP: Left anterior fascicle pacing; RWPT: R wave peak time; NS-LBBP: non-selective left bundle branch pacing; LVSP: left ventricle septal pacing; S-LBBP: selective left bundle branch pacing; ΔV6-RWPT NS-LBBP to LVSP: V6-RWPT shortening between the transition of NS-LBBP to LVSP
